# Evaluating the impact of domestication and captivity on the horse gut microbiome

**DOI:** 10.1038/s41598-017-15375-9

**Published:** 2017-11-14

**Authors:** Jessica L. Metcalf, Se Jin Song, James T. Morton, Sophie Weiss, Andaine Seguin-Orlando, Frédéric Joly, Claudia Feh, Pierre Taberlet, Eric Coissac, Amnon Amir, Eske Willerslev, Rob Knight, Valerie McKenzie, Ludovic Orlando

**Affiliations:** 1 Department of Animal Sciences, Colorado State University, Fort Collins, CO USA; 2 Department of Pediatrics, University of California San Diego, San Diego, CA USA; 3 Department of Ecology and Evolutionary Biology, University of Colorado, Boulder, USA; 4 Department of Computer Science and Engineering, University of California San Diego, La Jolla, California USA; 5 Department of Chemical and Biological Engineering, University of Colorado at Boulder, Boulder, CO 80309 Colorado USA; 6 National High-Throughput DNA Sequencing Center, University of Copenhagen, Øster Farimagsgade 2D entrance E, 1353 K Copenhagen, Denmark; 7 Centre for GeoGenetics, Natural History Museum of Denmark, University of Copenhagen, Øster Voldade 5-7, 1350 K Copenhagen, Denmark; 8 Association pour le cheval de Przewalski: TAKH, Station biologique de la Tour du Valat, 13200 Arles, France; 9 Laboratoire d’Ecologie Alpine (LECA), Centre National de la Recherche Scientifique and Université Grenoble-Alpes, Grenoble, France; 10 Center for Microbiome Innovation, University of California San Diego, La Jolla, California USA; 11 Laboratoire d’Anthropobiologie Moléculaire et d’Imagerie de Synthèse (AMIS), CNRS UMR 5288, Université de Toulouse, Université Paul Sabatier, 31000 Toulouse, France

## Abstract

The mammal gut microbiome, which includes host microbes and their respective genes, is now recognized as an essential second genome that provides critical functions to the host. In humans, studies have revealed that lifestyle strongly influences the composition and diversity of the gastrointestinal microbiome. We hypothesized that these trends in humans may be paralleled in mammals subjected to anthropogenic forces such as domestication and captivity, in which diets and natural life histories are often greatly modified. We investigated fecal microbiomes of Przewalski’s horse (PH; *Equus ferus przewalskii*), the only horses alive today not successfully domesticated by humans, and herded, domestic horse (*E. f. caballus*) living in adjacent natural grasslands. We discovered PH fecal microbiomes hosted a distinct and more diverse community of bacteria compared to domestic horses, which is likely partly explained by different plant diets as revealed by trnL maker data. Within the PH population, four individuals were born in captivity in European zoos and hosted a strikingly low diversity of fecal microbiota compared to individuals born in natural reserves in France and Mongolia. These results suggest that anthropogenic forces can dramatically reshape equid gastrointestinal microbiomes, which has broader implications for the conservation management of endangered mammals.

## Introduction

The gut microbiota of mammals provides the host with important functions, such as training the immune system early in life, metabolism, and synthesis of vitamins. This essential relationship between mammals and their microbes is the result of millions of years of co-evolution^1^. Therefore, disruptions between mammals and their microbial partners likely have major consequences for host health. In humans, the transition from hunter-gathering to farming and later to urban lifestyles included major dietary shifts, increased exposure to indoor environments, and the introduction of antibiotics and possibility of cesarean birth. These transitions are associated with a distinct and less diverse gut microbiome^2–6^ that has been implicated in the rise of immunologic and metabolic diseases^7–9^. A disruption of tightly evolved host-microbe relationships may be evident in non-human mammals as well, particularly those exposed to major diet and life history changes. We hypothesize that anthropogenic forces on mammals such as domestication and captivity in zoos may have reshaped mammal gut microbiomes. Domestication of animals is associated with modifications of species movement, feeding, protection, environment, and breeding, particularly to control behavior^10^. Captivity of mammals in zoos results in similar lifestyle modifications as domestication, but on shorter timescales and often with more extreme changes such as highly restricted diets, small habitat sizes, reduced social interactions, and higher exposures to antibiotics. Similar to humans, it is possible that these modifications will result in a distinct and less diverse gut microbiota in domesticated and captive mammals compared to wild conspecifics.

We investigated the effects of domestication and captivity on the fecal microbiomes, as a proxy for the gastrointestinal microbiome, of a unique pair of horse populations living in Mongolia. PH, which were never successfully domesticated, are enclosed by a fence to separate them from herded, domestic horse (Figure S1). Twenty-two individuals of PH were reintroduced in 2004 and 2005 to the 140 km^2^ reserve in Seer, Mongolia, and the population has grown to 59 individuals. Translocated animals originated from a 400 ha reserve in Villaret, France as well as the Prague zoo in the Czech Republic and the Kosice Zoo in Slovakia. Domestic horses live within a short range of the reserve on the Khomyn Tal plateau, which includes similar grazing vegetation as the Seer PH reserve (Figure S1).

Adult mammal gut microbiomes are largely constrained by host phylogeny^11,12^, but also influenced by diet^11,12^, cohabitation^13^, and host kinship to a lesser extent^14^. We acknowledge that care should be taken when comparing trends across mammals with very different digestive physiologies. However, we suspect that forces shaping gut microbiomes may be similar across different gastrointestinal physiologies because the gut microbiome serves the same basic function across mammals - providing the host with expanded metabolic capabilities such as additional energy and nutrient extraction from their diet^11^. Therefore, we hypothesized that PH and domesticated horse may differ in their gut microbiomes reflecting their divergence time of approximately 45,000 years ago^15,16^. Alternatively, because pedigree records and whole-genome sequence data show that all 2,100 PH individuals living today descend from approximately 12–16 founders including several domestic Mongolian mares^15,17–19^, PH may not host a distinct gut microbiome. We also hypothesized that the gut microbiomes of domesticated horses may be less phylogenetically diverse compared to PH, following microbiome diversity trends associated with humans living traditional versus urban lifestyles^2,4,6^. Alternatively, PH microbiomes may be less diverse due to extreme host genetic bottlenecks since the second half of the 19th century^15^. Additionally, we hypothesized that the two horse populations likely consumed a fairly similar plant diet because of their close proximity and similar forage environment. However, it is also possible that the two groups of horses consume different plant diets, which would likely contribute to differences in their fecal microbiomes. Finally, within the PH population, we hypothesized that horses born in zoo environments may be exposed to a different and less diverse suite of microbes^20^ that may persist even after reintroductions into reserves.

To test these hypotheses, we characterized fecal microbiomes via 16S rRNA amplicon sequencing of 44 PH and 28 domestic horses. In horses, fecal microbiomes are highly similar to the microbiome of the dorsal colon, but include many of the microbial taxa present in the ventral colon and cecum^21^, in which the bulk of fiber fermentation takes place. Therefore, we conclude that horse feces are an excellent, non-invasive means to assess the microbial fermentation communities in the large intestine of the horse. Additionally, we investigated whether horse populations were eating similar plant diets by amplicon sequencing of the P6 loop of the chloroplast *trn*L intron^22^ from the same fecal DNA extracts.

## Results and Discussion

**Figure 1:**
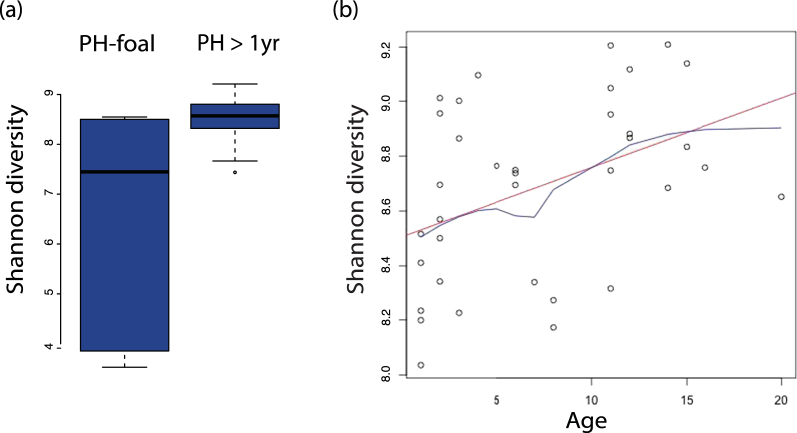
Age is an important influence on horse fecal microbiomes. (**a**) Shannon diversity of PH foals vs PH greater than 1 year of age (p = 0.0206). The five newborn foals exhibited great variation in Shannon diversity compared to older PH individuals. (**b**) Correlation of Shannon diversity with PH age (Spearman rho = 0.45, p = 0.0061). The red line indicates regression and the blue line a Lowess fit.

As in comparative studies of fecal microbiomes from human populations^6^, age had the most striking effect on horse fecal microbiome composition and diversity (Fig. 1, Figure S2). In horses >1 year in age, fecal microbiomes of both horse populations were dominated by taxa in the groups Bacteroidales, Treponema, Bacteroidetes, Fibrobacter, and Lachnospiraceae (Figure S3). The PH population included five pre-weaned newborn foals, which had a less phylogenetically diverse (Fig. 1a), and compositionally distinct (Figure S3) fecal microbial community compared to PHs over the age of 1 year. Two of the five PH foals had fecal microbiomes with very high abundance of Bacteroides (Figure S3), which are typically in high abundance before weaning in mammals^23^. The correlation of fecal microbial diversity present within the PH population and age remained significant even with foals excluded (Fig. 1b).

**Figure 2:**
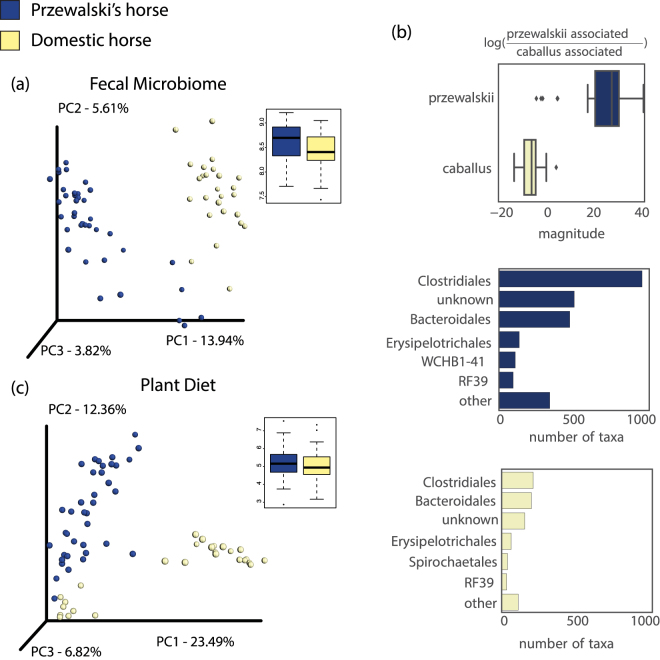
Diversity and composition of PH and domesticated horse fecal microbiomes and diets (foals excluded). (**a**) PCoA plot based on 16S rRNA amplicon unweighted UniFrac distances (host population; r^2^ = 0.12, Pr(>F) = 0.001), and box plots showing 16S rRNA Shannon diversity of PH and domesticated horse individuals (p = 0.0356). (**b**) Principal balances were constructed to quantify the microbial community shifts with respect to horse lineage. The balance representing the microbial differences between PH and domestic horse defines a partition of microbes that are associated with each lineage. Specifically, these results revealed 2599 PH-associated microbial taxa strongly associated with PHs and 803 with domesticated horses with an uncorrected p-value of 7.40 × 10^−21^. (**c**) PCoA plot based on trnL Bray Curtis distances (host population; r^2^ = 0.14, Pr(>F) = 0.001), and box plots showing trnL Shannon diversity (p = 0.3283).

PH fecal microbiomes differ in composition and are more diverse compared to domesticated horses (Fig. 2). The two horse populations’ fecal microbiomes were phylogenetically distinct (Fig. 2a), driven by different ratios of taxa in the Orders Clostridiales, Bacteroidales, Erysipelotrichales, and Spirochaetales (Fig. 2b). An Analysis of Composition of Microbiomes (ANCOM) revealed that PH fecal microbiomes had a significantly higher abundance of the Clostridia genus *Phascolarctobacterium* (Table S1), which is associated with the succinate pathway for producing the short chain fatty acid propionate^24^. Furthermore, PH fecal microbiomes harbored a significantly higher relative abundance of Archeal methanogen *Methanocorpusculum*. While these differences may be suggestive of PH and domestic horse gut microbiomes utilizing different gut metabolism pathways, we also found that individuals from the two horse lineages consumed a similar level of plant diversity, yet differing compositions of plant taxa, as indicated by trnL gene maker molecular operational taxonomic units (MOTU) (Fig. 2c, Figure S4).

Diet differences likely account for some of the differences in gut microbiota, as demonstrated by a significant but weak correlation via a Procrustes Analysis of 16S rRNA and trnL Principal Coordinate Analysis (PCoA) plots as well as a Mantel test on 16S rRNA and trnL distance matrices (Figure S5). Overall, the diet of PH contained many plant MOTUs that were either not detected or present at lower abundance in domestic horse (Table S2, Figure S6). This included MOTUs with taxonomy matching the thorny shrub family Elaeagnaceae such as sea buckthorn (*Hippophae*). Sea buckthorn has been demonstrated to be rich in vitamins, carotene, flavonoids, essential oil, carbohydrates, organic acids, amino acids, and minerals^25^, and was originally named “hippo” (horse) “phaos” (glossy) because it was believed to give horses a shiny coat^26^. Also more abundant in PH were the grasses *Stipa glareosa/gobica*, locally known for their high palatability and protein content^27^, and trees of the genus *Salix* which is known, for some species, for its antioxidant, antimicrobial and cytotoxic properties^28,29^. These dietary differences may be due to the fact that the rugged, ecologically diverse PH enclosure hosts 62% of the whole Khomyn Tal plant diversity, although accounting for only 5% of its area^30^. For example, sea buckthorn and *Salix* species were not observed in the range of the domestic horses. Grazing preferences and human influences on horse movements may be additional contributing factors and further research is required to assess the relative weights of all drivers.

PH individual fecal microbiomes had a significantly more diverse consortia of bacteria compared to domestic horses (Fig. 2a), although inter-individual variation was lower (Figure S7). It is possible that the distinct PH diet supports a higher diversity of gastrointestinal microorganisms. Another possibility is that gut diversity in horses was lost as a result of domestication, mirroring losses detected in humans transitioning to agricultural and urban lifestyles. Furthermore, we discovered that inter-individual variation was significantly lower in the PH population compared to the domestic population (Figure S7), which is similar to a trend discovered in gut microbiomes of a small human hunter-gatherer population in South America^2^. Whether this trend is due to a less diverse host genetic pool or due to similar diets within the small host population is unknown. Testing these various intriguing hypotheses would require comparisons of additional equine populations, as well as comparisons of other pairs of wild and domesticated animals.

**Figure 3:**
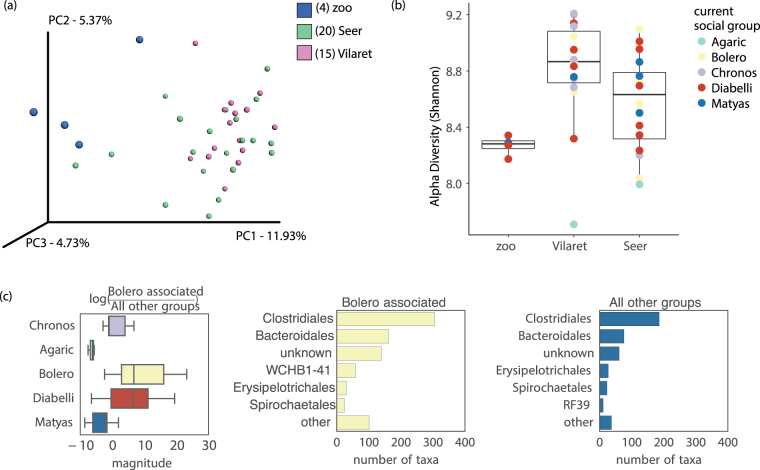
Life history shapes PH microbiomes. (**a**) PCoA plot of an unweighted UniFrac distance matrix of PH 16S rRNA data. Fecal microbiome samples representing horses born in European zoos are shown as large blue spheres. (**b**) Shannon diversity by location of birth with individual data points colored by social group. Zoo-born individuals’ fecal microbiomes were significantly lower alpha diversity than individuals born in a reserve in Vilaret, France. The lowest diversity sample (Agaric social group, born in Vilaret), is from a 19-year-old individual, the second oldest in the data set. (**c**) Principal balances were constructed to determine whether particular consortia of fecal microbes associated with PH individuals in the same social group. A notable difference was detected between the Bolero (818 associated) and Matyas social groups (421 associated) with an uncorrected p-value of 8.8 × 10^−04^.

PH fecal microbiomes are influenced by life history. The PH population in Seer Mongolia is composed of individuals born in several locations. Of the 39 non-foals, 20 were born in the Seer reserve itself, and 19 were relocated in 2004–2005 to the reserve after birth in Villaret, France (n = 15) and in 2011 from European zoos (N = 4). We discovered that place of birth was associated with significant although small differences in fecal microbiota, even after taking into account social group membership (r^2^ = 0.093, Pr(>F) = 0.001). In particular, the four PH individuals born in the European zoos appeared to harbor a distinct (Fig. 3a) and less diverse community of microbes (vs Villaret p = 0.016, vs Seer p = 0.33, Fig. 3b). In contrast, the animals born in Villaret, a nature reserve, harbor a community that is more similar in composition to those born in Seer. We first considered that these trends in fecal microbiome diversity may be related to diet diversity. However, fecal microbial diversity was only weakly correlated with the plant diversity found in their feces (Spearman rho = 0.075, p = 0.05), suggesting that current diet diversity may not necessarily equate to a diverse gut community. Furthermore, diversity of diet was not associated with place of birth (Figure S8). Therefore, we hypothesize that birth environment is another factor important in shaping gut microbiomes of PHs. We may be witnessing potential lasting founder effects of colonization by microbes in early life. In a similar vein, human infants born in differing environments (cesarean vs vaginal delivery) assemble different starting gut microbiomes, with differences persisting for over a year in some cases^31,32^.

PHs are social animals, with families consisting of a single dominant stallion bonding with adult females over many years, and their common offspring who stay in their natal group often beyond puberty. We compared fecal microbiomes collected from PH individuals (foals excluded) and found that social structure has a significant effect on the composition of horse fecal microbiomes (r^2^ = 0.13, Pr(>F) = 0.005). To remove the potential influence of relatedness within social group, we also included a test with only reproductive adults (r^2^ = 0.14, Pr(>F) = 0.058). Similar to fecal microbiome taxa differentiating host lineages, taxa in Orders Clostridiales, Bacteroidales, Erysipelotrichales, and Spirochaetales differed among social groups (Fig. 3c). Fecal microbiome similarities within social groups could be due to a shared similar diet as individuals belonging to the same social groups tend to graze together and tend to share a more similar diet profile than expected by chance (r^2^ = 0.18, Pr(>F) = 0.001). In this study, we cannot thus disentangle the effects of diet and social contacts. However, in wild baboon populations, Tung *et al*.^33^ demonstrated that rates of social interactions influenced the taxonomic structure of gut microbiomes when controlling for diet, kinship, and shared environments. Therefore, it is likely that herd social structure is important for shaping gastrointestinal microbiomes of PHs, and other social mammals. Disruption of social interactions is one of several ways in which anthropogenic forces such as captivity may influence mammal gut microbiomes.

Finally, we investigated the influence of horse relatedness on shaping PH fecal microbiomes, and discovered a significant correlation between kinship (most similar = 1) and microbiome composition (most similar = 0) (Mantel r = −0.34, p = 0.002). We focused on reproductive adults for these correlations to remove the influence of social group in kinship, which for reproductive adults are not correlated (r^2^ = 0.12, Pr(>F) = 0.54). However, we are limited in disentangling all possible confounding variables, such birth location, by the small sample size in this data set.

We are only beginning to understand the consequences of disrupting relationships between mammals and their microbial partners that have evolved over tens of millions of years. Domestication, as already shown for captivity^34^, may result in disruptions of gut microbial communities, especially in cases with diet shifts and restricted exposure to outdoor environments. This is particularly important for conservation managers as they urgently try to slow the Earth’s sixth major extinction in the wild. Therefore, it is critical to document the variables influencing the structure and diversity of microbiomes in wildlife populations with an eventual goal of considering microbiome health when managing endangered populations. Our findings also highlight the importance of life history in shaping the horse gastrointestinal microbiome. Importantly, a legacy of captivity may persist in zoo-born individuals, which we discovered harbored a much lower gut diversity than reserve-born individuals, with potential long-term survival consequences that may impact the outcome of reintroduction programs. Additional research on other zoo-born, re-introduced mammals is warranted. Finally, although some domesticated livestock have extinct wild ancestors (e.g. cattle), many still exist and can be studied. We suggest this could be a fruitful endeavor with possible health implications for the agricultural industry.

## Methods

### Sample collection

The PH population in Seer, Mongolia is intensely observed daily by the rangers of the Association TAKH. As a result, each horse is identifiable and associated with detailed life history data, including pedigree, age, social structure, health, and years of daily behavioral observations in the field (Table S3). Relatedness coefficients (Table S4) were calculated based on the known pedigree of each individual PH as reported in the international PH studbook, and using the tabular method^35^.

Fresh fecal samples were collected from PH over seven days using binoculars to associate each fecal sample to an individual horse. Each horse had 1–4 samples collected over 1–3 days between 6/2/2014–6/9/2014. Each fecal sample collected was geolocated using a GPS device (Table S5) and subsampled for storage in both RNAlater and 95% ethanol, within 2–38 minutes after defecation (median = 7 minutes). In many cases, technical replicates of fecal samples were collected by subsampling feces for storage in multiple RNAlater and 95% ethanol aliquots (Table S5). Samples were stored at + 4 C in a transportable coolbox within 4–93 minutes post-sampling (median = 24 minutes) (Table S5). The same sampling procedure was applied to domestic horses living within a short range of the Seer reserve and on the Khomyn Tal plateau, except that *(i)* sampling lasted over two days, *(ii)* sampling was performed within 1–80 minutes after defecation (median = 18 minutes), and *(iii)* samples were stored at + 4 C within 1–74 minutes post-sampling (median = 16 minutes) (Table S5). We also report horse spatial proximities that were estimated by recording the horses’ nearest neighbor(s) (nnjj1, nnjj2, nnjj3, Table S3) within 2 hours of daily observations from January 1^st^ to June 10^th^ 2014, disregarding animals involved in less than 100 hours of observations, and normalizing by the total number of observations available per individual.

All methods were carried out in accordance with relevant guidelines and regulations. Because fecal material was collected without interacting with horses directly, approval for experimental protocols on vertebrate animals was not required. A CITES permit for the export of fecal material was granted (Certificate No. 1000543).

### DNA extraction

Fecal samples were initially transported from Seer, Mongolia to Centre for GeoGenetics in Copenhagen, Denmark. Subsequently they were shipped to University of Colorado Boulder, where DNA was extracted using the PowerSoil-htp 96 Well Soil DNA Isolation Kit following Earth Microbiome Project (EMP) standard protocols (http://www.earthmicrobiome.org/protocols-and-standards/dna-extraction-protocol/). This DNA extraction method is standard for both the Human Microbiome Project (https://www.hmpdacc.org/hmp/doc/HMP_MOP_Version12_0_072910.pdf) and the EMP, and has been demonstrated as robust for recovering microbial DNA from fecal samples^36^.

### 16S rRNA amplicon data generation

Subsequently, we amplified the V4 region of the 16S rRNA gene with the 515 f/806r primer set that included golay error-correcting barcodes on the forward primer per EMP protocols (http://www.earthmicrobiome.org/protocols-and-standards/16 s/). Amplicons were sequenced on a HiSeq 2500 on rapid run mode at the University of California San Diego Institute for Genomic Medicine Genomics Center. Forward sequence reads (~125 nucleotides) were demultiplexed using QIIME version 1.9^37^. The forward read sequences (125 bp) were clustered using a sub-operational-taxonomic-unit (sOTU) approach, which uses error profiles to improve accuracy and specificity of sequences (i.e. correctly identify true single nucleotide polymorphisms versus Illumina platform sequencing errors that occur at a rate of 0.1% per nucleotide)^38^. sOTUs present in the data set less than 25 times (~0.0005) were filtered out, along with sequences identified as chloroplast and mitochondria. Samples were then rarified to 30,000 sequences per sample for downstream analyses.

### Diet data generation

We used the DNA extracts prepared for 16S rRNA sequencing to investigate fecal plant DNA content. We utilized a metabarcoding approach in which the P6 loop of the *trn*L (UAA) intron of chloroplast DNA (primers g and h; Taberlet *et al*.)^22^ was targeted by PCR, following the procedures described in Valentini *et al*. (2009) and performing PCR amplifications in four replicates. PCR amplifications were performed on 384 wells plates and contained 1x AmpliTaq Gold^®^ 360 Mastermix (Life Technologies), 0.5 µM of each primer, 0.0032 mg bovine serum albumin (BSA, Roche Diagnostics) and 2 µL DNA extract, in 20 µL reaction volume. Thermocycling conditions had an initial denaturation step of 10 minutes at 95 °C, followed by 40 cycles of 30 seconds at 95 °C, 30 seconds at 50 °C, and 60 seconds at 72 °C. Each reaction was tagged by a unique combination of primers^39^ containing each one index of 8 nucleotides (with at least 5 differences among them), allowing sequence-demultiplexing after sequencing. PCR products were purified using the MinElute PCR purification kit (QIAGEN GmbH), and finally mixed together using the same volume per reaction. Library preparation and sequencing were performed at Fasteris facilities (Geneva, Switzerland). Libraries were prepared using the MetaFast protocol (www.fasteris.com/metafast) and a pair-end sequencing (2 × 125 bp) was carried out using an Illumina HiSeq 2500 sequencer (Illumina, San Diego, CA, USA) using the HiSeq SBS Kit v4 (Illumina, San Diego, CA, USA) following the manufacturer’s instructions. Sequence demultiplexing, trimming and filtering was performed using obitools^22,39,40^, removing trimmed sequences shorter than 10 bp and represented by less than 100 sequence reads over the whole data set. MOTU identification was done using the ecoTag algorithm^40^, the reference database was constituted from the EMBL Nucleotide Sequence database release 126^41^ using ecoPCR^42^.

### Quantification and Statistical Analysis

#### Diversity estimates and statistical approaches

We estimated alpha diversity for 16S rRNA and trnL data using the Shannon diversity index. We tested for significant effects of categorical metadata for alpha diversity using a Kruskal-Wallis test and report a Bonferroni corrected p-value. We tested Spearman correlations between numerical metadata/data categories and report a Rho and p-value using R software^43^. Beta diversity was assessed for 16S rRNA data using the unweighted UniFrac phylogenetic metric^44,45^ and for trnL data using the Bray Curtis metric. Principal coordinates analysis (PCoA) was run on the resulting distance matrices and the resulting coordinates were visualized using Emperor 0.9.5^46^. We tested for significant differences between categories for beta diversity using a PERMANOVA (Adonis) with a p-value based on 999 permutations (R, vegan package)^43^. We tested for correlations between distance matrices (e.g. 16S rRNA and trnL) using a mantel test and report a mantel r and a p-value based on 999 permutations. We compared PCoA plots using a Procrustes analysis, which transforms two principal coordinates (PCs) by rotating, scaling, and translating to minimize distance between matching samples. We report an M^2^ value, which is an estimate of distance between matched points, and a p-value calculated by shuffling identifiers in one PC 999 times and recalculating M^2^ values. These analyses were run using QIIME 1.9^37^. We compared within and between horse population beta diversity distances using a two-sided Student’s two-sample t-test and report a Bonferroni-corrected p-values calculate using 999 permutations with QIIME 1.9^37^.

Principal balances^47^ were constructed to quantify the microbial community shifts with respect to horse lineage, age and social group. The principal balances were constructed from applying the isometric log-ratio transform to a tree generated from Ward hierarchical clustering of the microbial abundances. Proportionality was used to define a distance metric to perform the hierarchically clustering, given by the following equation.

$$d(xi,xj)=\,\mathrm{ln}[\frac{xi}{xj}]$$

where *xi*, *xj* refers the proportions of microbes *i* and *j*.

Using this procedure can construct a tree where highly correlated microbes are clustered together in a greedy fashion. With these balances, a linear regression was used to correlate the balances against horse lineage, age and social group^48^.

The Analysis of Composition of Microbiomes (ANCOM) procedure compares the relative abundance of a taxon between two ecosystems by computing Aitchison’s^49^ log-ratio of abundance of each taxon relative to the abundance of all remaining taxa one at a time. Thus, if there are “m” taxa, then for each taxon it performs “m-1” tests and the significance of each test is determined using the Benjamini-Hochberg procedure that controls for FDR at 0.05. To deal with the zero counts, we used an arbitrary pseudocount of 1. For a more detailed description of ANCOM we refer the reader to Mandal *et al*.^50^.

### Assessing potential technical artefacts

We investigated potential technical artefacts related to collecting samples in a remote field site that may influence microbiome diversity. These technical variables include method of sample preservation (PH and domestic), sample variation over days (PH), delay between defecation and sampling (PH), and delay between sampling and cooling (PH).

Fecal microbial communities can change during storage, depending on conditions such as preservative and temperature^51^. This poses significant challenges for microbiome studies that require remote field campaigns for sample collection. Based on previous research^51^, we chose two robust preservatives, RNAlater and 95% ethanol for storage of fecal samples to help reduce microbial community change during sample storage and transport. We compared fecal microbial and plant community composition and diversity in each preservation type (Figures S2 and S4).

Because the preservation methods of RNAlater and 95% ethanol significantly affected microbiome composition and diversity (Figures S2 and S4), data was split based on preservation method for further analyses. For the main text set analyses, we utilized data generated from samples stored in RNAlater because they included a higher diversity of both microbes and plant DNA.

We next investigated the effect of delay between defecation (delay1) and sampling and between sampling and cooling (delay2) discovered that neither delay significantly affected microbiome compositions when individual horse is controlled (RNAlater: delay1- F_1,65_ = 0.99, r^2^ = 0.01, p = 0.4265, delay2 - F_1,65_ = 0.87, r^2^ = 0.01, p = 0.7485; ethanol: delay1- F_1,15_ = 0.99, r^2^ = 0.04, p = 0.4926, delay2 - F_1,15_ = 0.96, r^2^ = 0.04, p = 0.5275).

Finally, we tested microbiome variability within PH by sampling feces from the same individuals over multiple days. We reduced the ethanol-preserved and RNAlater-preserved sample sets to include only samples from individuals with replicate samples. We then calculated distances within fecal sample replicates and across multiple days. A PERMANOVA on unweighted UniFrac distances for replicate samples stored in RNAlater revealed that individual horse was highly significant (F_29,65_ = 2.55, r^2^ = 0.67, p < 0.0001) and day of sampling less so when controlling for individual horse (F_6,65_ = 2.55, r^2^ = 0.06, p = 0.0334). For ethanol-preserved samples with replicates, individual horse was highly significant (F_7,15_ = 2.71, r^2^ = 0.69, p < 0.0001) and day of sampling was not significant when controlling for individual horse (F_3,15_ = 1.12, r^2^ = 0.12, p = 0.2103), although we note our power is lower in the ethanol group (n = 16, vs n = 66 in the RNAlater group).

To generate a single representative sample for each individual PH, sOTUs were summed using the QIIME script collapse_samples.py. We focus analyses of the sample set preserved in RNAlater as noted above.

### Data Availability

16S rRNA raw sequence data have been deposited in QIITA study under accession 10171 and EBI under accession ERP016897. trnL data have been deposited in Dryad Digital Repository 10.5061/dryad.kc7h9.

## Electronic supplementary material

Supplementary Information

Table S1

Table S2

Table S3

Table S4

Table S5
