# Supplementary material for: Evaluating the impact of domestication and captivity on the horse gut microbiome
Source: Sci Rep. 2017 Nov 14;7:15497. doi: 10.1038/s41598-017-15375-9 (PMC5686199; doi:10.1038/s41598-017-15375-9)
Supplement: Supplementary file 1 — Supplementary Information [file 41598_2017_15375_MOESM1_ESM.pdf]

**Supplemental Information for**

# Evaluating the impact of domestication and captivity on the horse gut microbiome

**Jessica L. Metcalf[1], Se Jin Song[2,3], James T. Morton[2,4], Sophie Weiss[5], Andaine Seguin-Orlando[6,7], Frédéric Joly[8], Claudia Feh[8], Pierre Taberlet[9], Eric Coissac[9], Amnon Amir[2], Eske Willerslev [7], Rob Knight[2,4,10], Valerie McKenzie[4], Ludovic Orlando[7,11*]**

1 - Department of Animal Science, Colorado State University, Fort Collins, CO, USA
2 - Department of Pediatrics, University of California San Diego, CA, USA
3 - Department of Ecology and Evolutionary Biology, University of Colorado, Boulder, USA
4- Department of Computer Science and Engineering, University of California San Diego, La Jolla, California, USA
5 - Department of Chemical and Biological Engineering, University of Colorado at Boulder, Boulder, CO 80309
6 - National High-Throughput DNA Sequencing Center, University of Copenhagen, Øster Farimagsgade 2D entrance E, 1353K Copenhagen, Denmark,
7 -Centre for GeoGenetics, Natural History Museum of Denmark, University of Copenhagen, Øster Voldade 5-7, 1350K Copenhagen, Denmark
8 - Association pour le cheval de Przewalski : TAKH, La Tour du Valat, 13200 Arles, France
9 - Laboratoire d'Ecologie Alpine (LECA), Centre National de la Recherche Scientifique and Université Grenoble-Alpes, Grenoble, France
10 – Center for Microbiome Innovation, University of California San Diego, La Jolla, California, USA
11 -Laboratoire d'Anthropobiologie Moléculaire et d'Imagerie de Synthèse (AMIS), CNRS UMR 5288, Université de Toulouse, Université Paul Sabatier, 31000 Toulouse, France

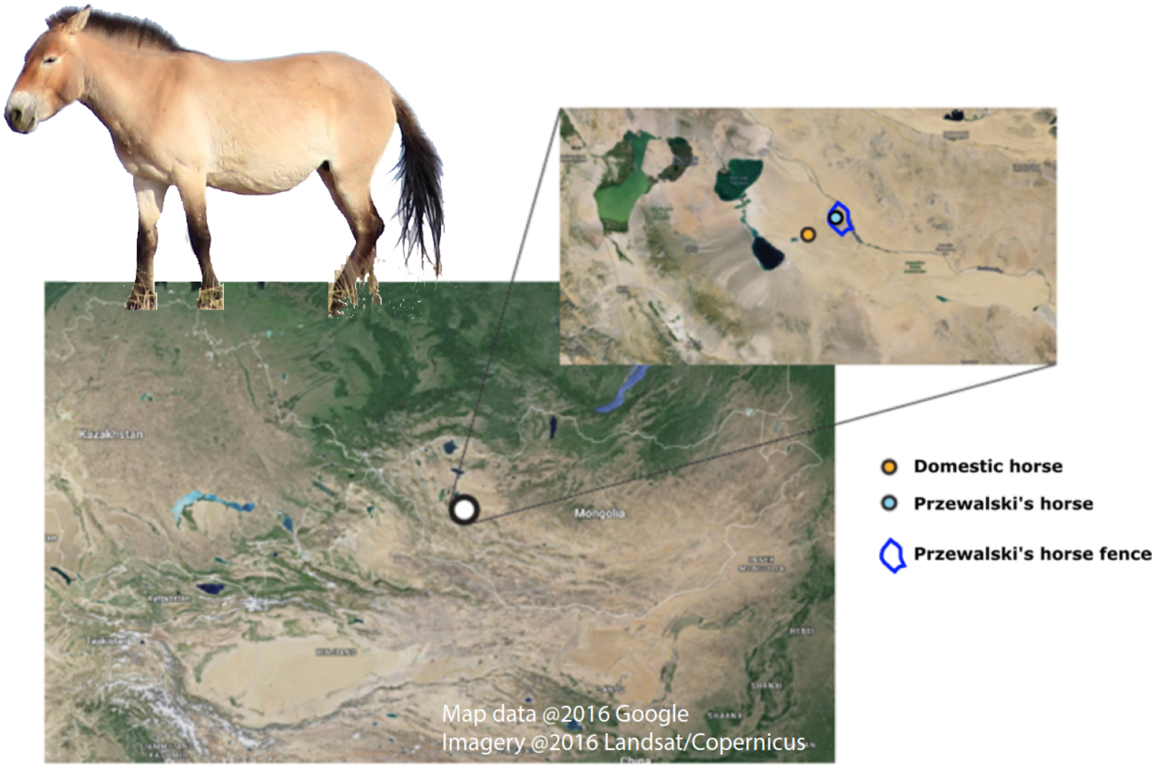


**Figure S1.** Przewalski's horses (PH) are characterized by a stocky build with short, darkly striped legs, and an erect dark mane. Feces of PH and free-roaming, domestic horses were collected from physically separated areas in the Khomyn Tal region of Mongolia (PH area is fenced). The PH population is located approximately at N 47.86 and E 94.14, and the Domestic horse is located approximately at N 47.76 and E 94.00. Photo by L. Orlando. Map data is from Google @2016 and Imagery @2016 Landsat/Copernicus

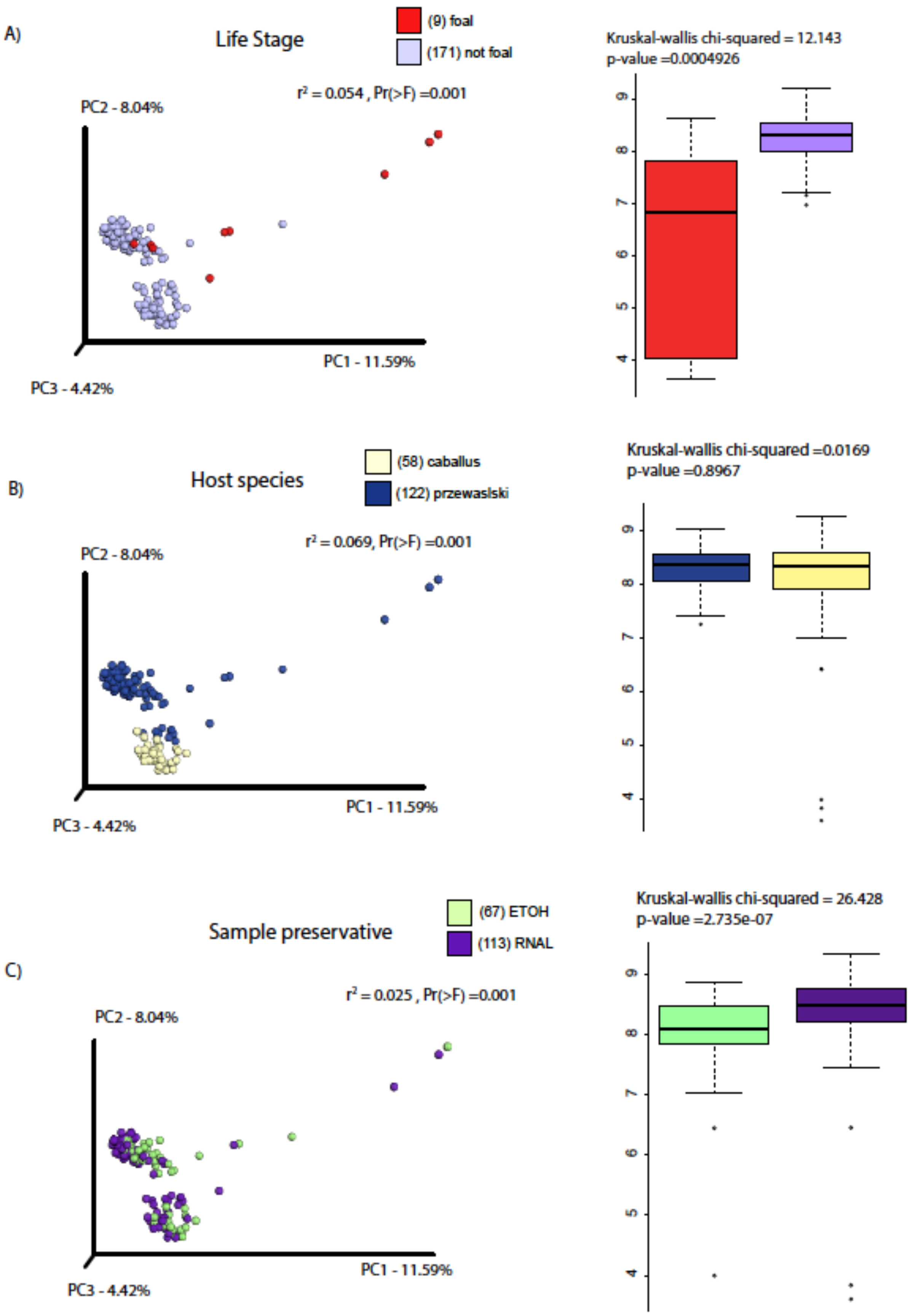
A)
Life Stage
(9) foal
(171) not foal
r² = 0.054 , Pr(>F) =0.001
PC2 - 8.04%
PC1 - 11.59%
PC3 - 4.42%
Kruskal-wallis chi-squared = 12.143
p-value =0.0004926
B)
Host species
(58) caballus
(122) przewaslski
r² = 0.069, Pr(>F) =0.001
PC2 - 8.04%
PC1 - 11.59%
PC3 - 4.42%
Kruskal-wallis chi-squared =0.0169
p-value =0.8967
C)
Sample preservative
(67) ETOH
(113) RNAL
r² = 0.025 , Pr(>F) =0.001
PC2 - 8.04%
PC1 - 11.59%
PC3 - 4.42%
Kruskal-wallis chi-squared = 26.428
p-value =2.735e-07

**Figure S2.** PCoA plot based on unweighted UniFrac 16S rRNA distance matrix and alpha diversity (Shannon) of all PH and domestic horse fecal samples, including fecal replicates and samples preserved in both ethanol and RNAlater. (A) Newborn foals had the largest differences in their composition of gut microbiota (PC1). Foals also had significantly lower Shannon diversity. (B) Gut microbiota differences between PH and domestic horse (PC2) were also significant, although Shannon diversity was only slightly higher in PH (note: this trend became significant once the data set was split by preservative and foals were removed). (C) Differences between microbiomes due to preservation in RNAlater versus 95% ethanol was also significant and visible on PC2. Additionally, RNAlater preserved a higher diversity of microbes. For each variable, we report results of a PERMANOVA test (beta diversity) and Kruskal-Wallis test (alpha diversity).

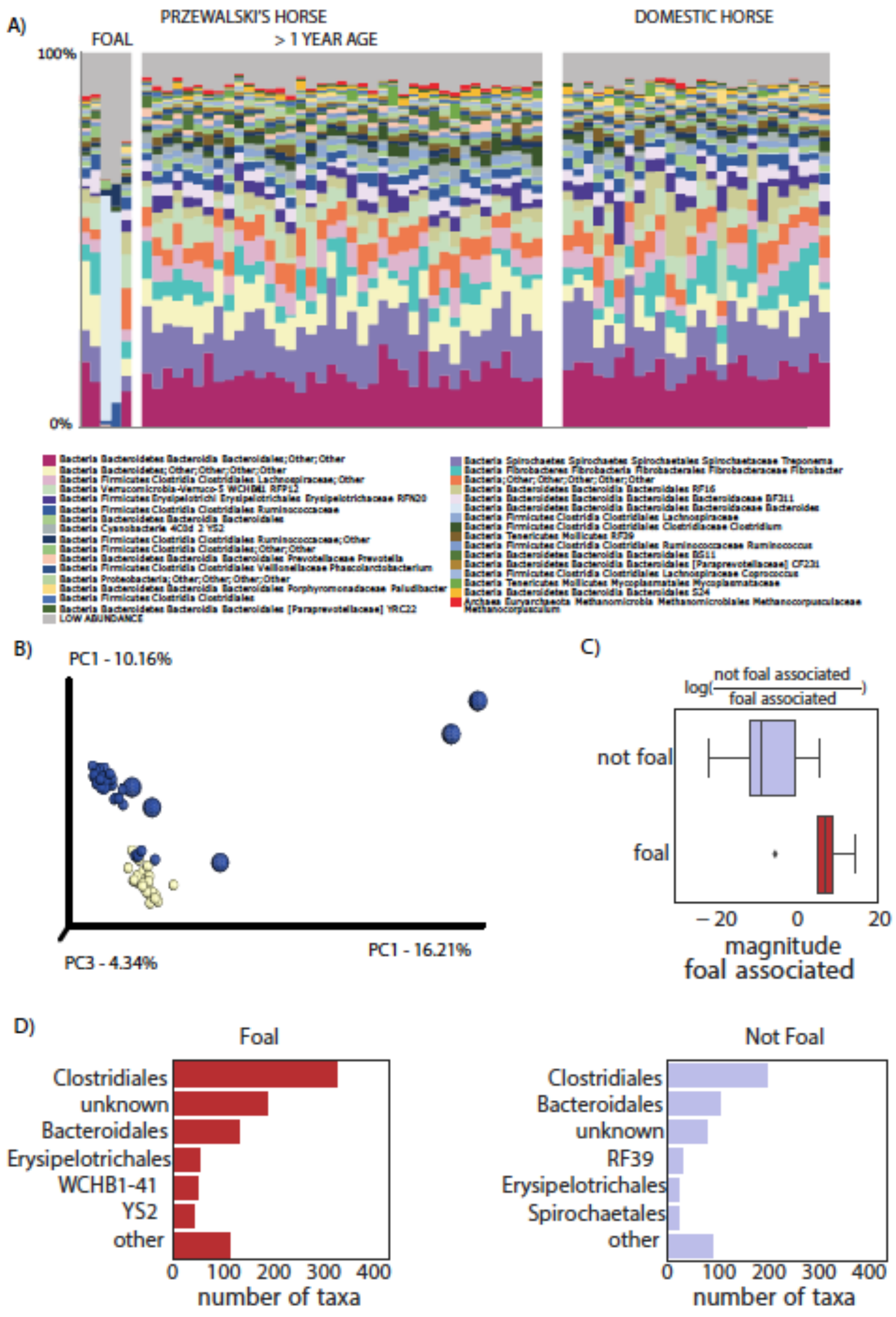


**Figure S3.** (A) Relative abundance of microbial taxa in 5 PH foals, 39 >1 year old PH, and 36 feral, domesticated horse (samples collapsed to one representative sample per individual) B) PCoA plot based on an unweighted UniFrac distance matrix of 5 PH foals (blue, large spheres), 39 PH individuals >1 year (blue), and 36 feral, domesticated horses (yellow) living in near Seer, Mongolia. (C) Principal balances were constructed to quantify microbial community differences between foal and non-foal PHs. The balance representing the microbial difference between foal and non-foal PHs defines a partition of microbes that are associated with each age group. (D) We discovered 837 foal associated taxa and 492 non-foal associated taxa.

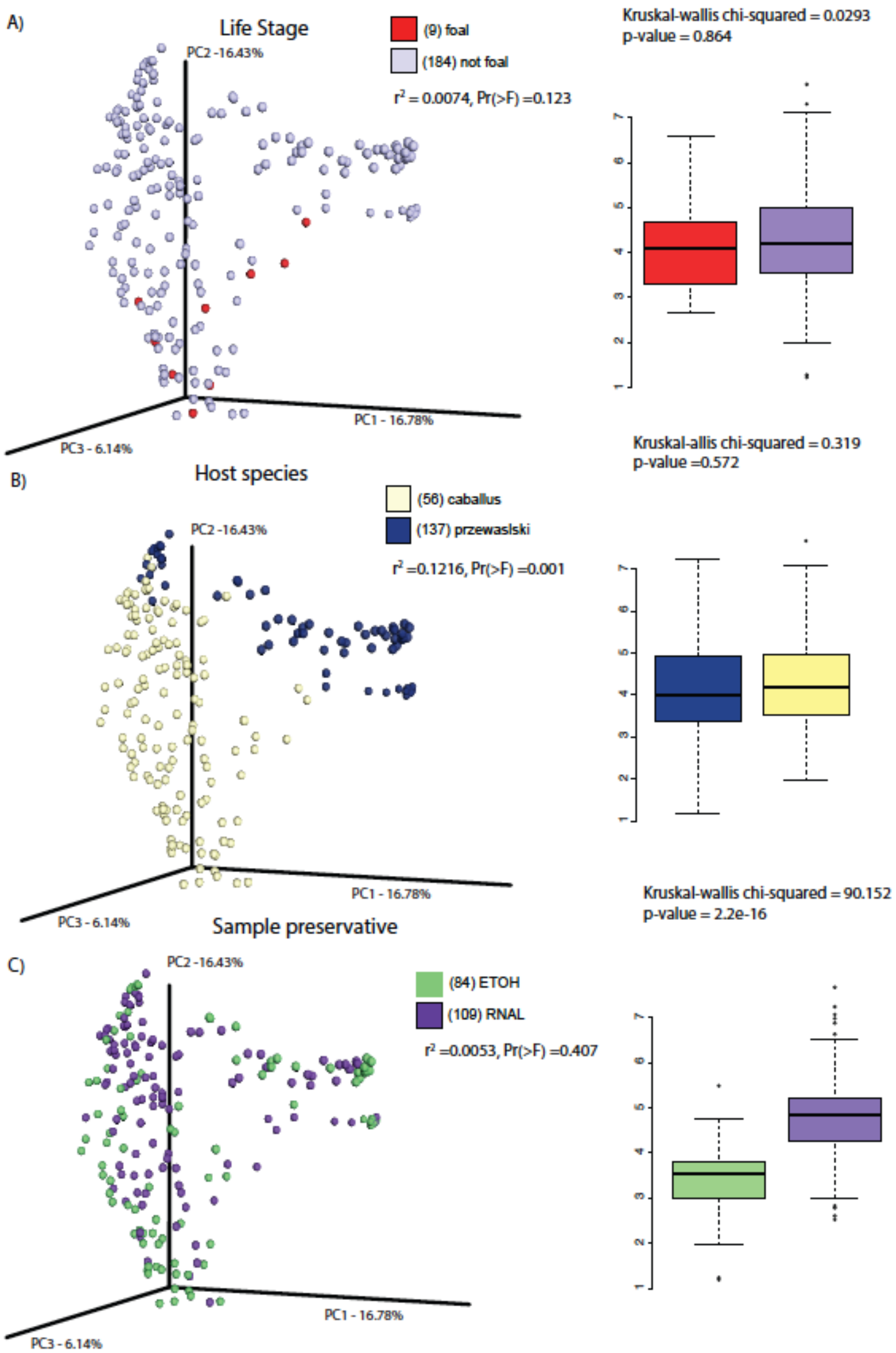
A)
Life Stage
(9) foal
(184) not foal
r² = 0.0074, Pr(>F) =0.123
PC2 -16.43%
PC1 - 16.78%
PC3 - 6.14%
Kruskal-wallis chi-squared = 0.0293
p-value = 0.864
B)
Host species
(56) caballus
(137) przewaslski
r² =0.1216, Pr(>F) =0.001
PC2 -16.43%
PC1 - 16.78%
PC3 - 6.14%
Kruskal-allis chi-squared = 0.319
p-value =0.572
C)
Sample preservative
(84) ETOH
(109) RNAL
r² =0.0053, Pr(>F) =0.407
PC2 -16.43%
PC1 - 16.78%
PC3 - 6.14%
Kruskal-wallis chi-squared = 90.152
p-value = 2.2e-16

**Figure S4.** (A) PCoA plot based Bray Curtis trnL distance matrix and alpha diversity (Shannon) of all PH and domestic horse individual fecal samples, including fecal replicates and samples preserved in both ethanol and RNAlater. Samples sizes for each treatment group are shown in parentheses. Newborn foals had significantly different composition plants in their fecal material than horses > 1 year of age, although Shannon diversity was similar between the two age groups. (B) The composition but not Shannon diversity differed between horse lineages. (C) Whether samples were preserved in RNAlater or 95% ethanol had a significant effect on both composition and Shannon diversity. Similar to 16S rRNA data, RNAlater preserved a higher diversity of plant material. Therefore, RNAlater data sets were used for downstream analyses. For each variable, we report results of an PERMANOVA test (beta diversity) and Kruskal-Wallis test (alpha diversity).

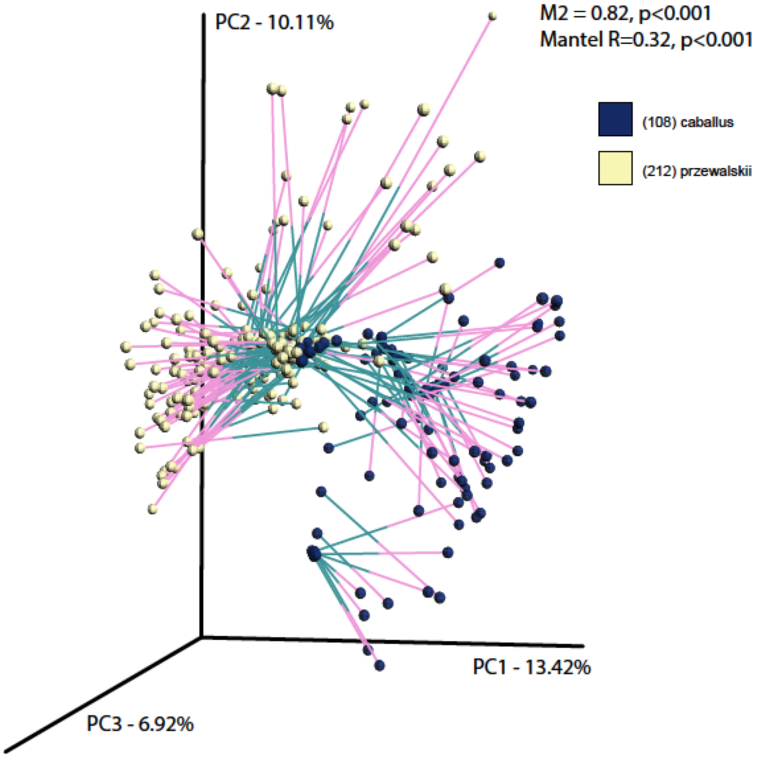


**Figure S5**. Procrustes analysis of principal coordinates of trnL and 16S rRNA data revealing a significant ($p<0.001$), but weak correlation ($M^2=0.82$) between PCoA patterns in microbiome and diet data sets. All fecal samples are shown (not collapsed to individual), including replicates and samples preserved in both ethanol and RNAlater. A Mantel test on distance matrices also revealed a significant correlation.

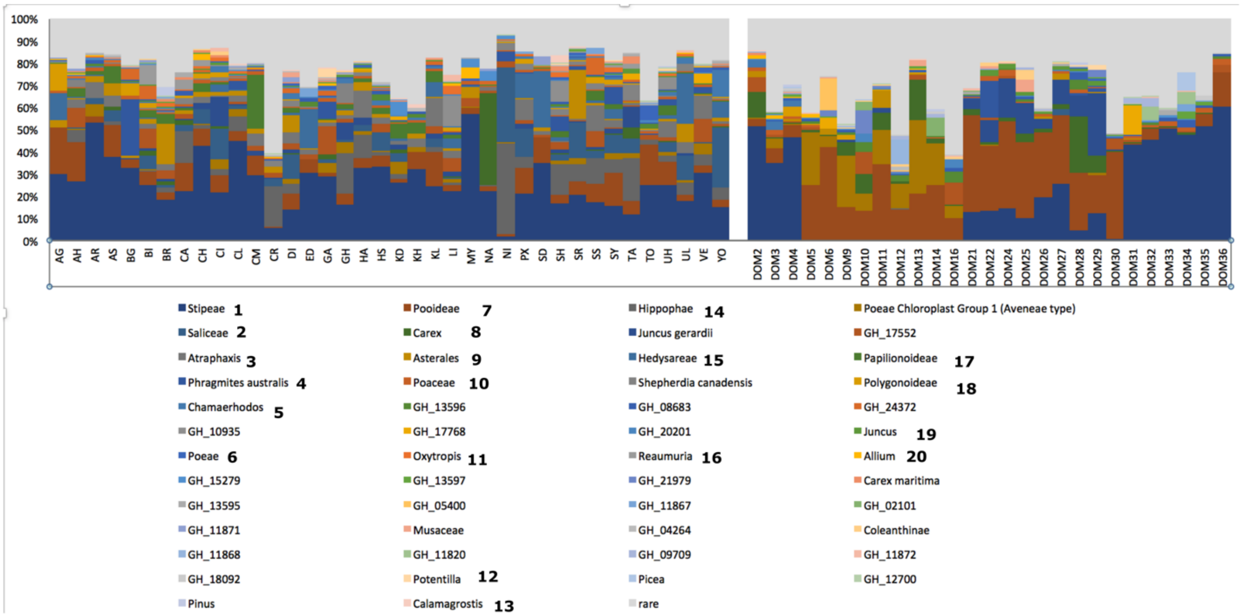


1: Stipa glareosa/gobica
2: Salix ledebouriana and Salix microstachya
3: Atraphaxis pungens
4: Phragmites australis
5: Chamaerhodos sabulosa
6: Agrostis mongolica
7 and 10: Any grass species
8:Carex stenophylloides, Carex duriuscula, Carex enervis and Carex orbicularis
9: Any Daisy like species
11: Oxytropis tragacanthoides, Oxytropis aciphylla, Oxytropis mongolica, Oxytropis salina
12: Potentilla anserina
13: Calamagrostis epigeios
14: Hippophae rhamnoides
15: Hedysarum fruticosum
16: Reaumuria soongorica
17: Any legume species
18: Polygonum sibiricum
19: Juncus salsuginosus
20: Allium mongolicum, Allium polyrrhizum, Allium anisopodium

**Figure S6.** Relative abundance of fecal plant taxa estimated using the trnL marker gene (samples collapsed to one representative sample per individual). OTUs for which taxonomy could not be assigned are shown as "GH_XXXX". Relative abundance of the 50 most abundant taxa in the entire data set are shown in colors according to the figure legend. All other taxa were summed and are shown as a light gray bar that represents all "rare" taxa. We also provide a reference of 20 plant taxa that have been collected from this region and match them to the plant group identified by the trnL data.

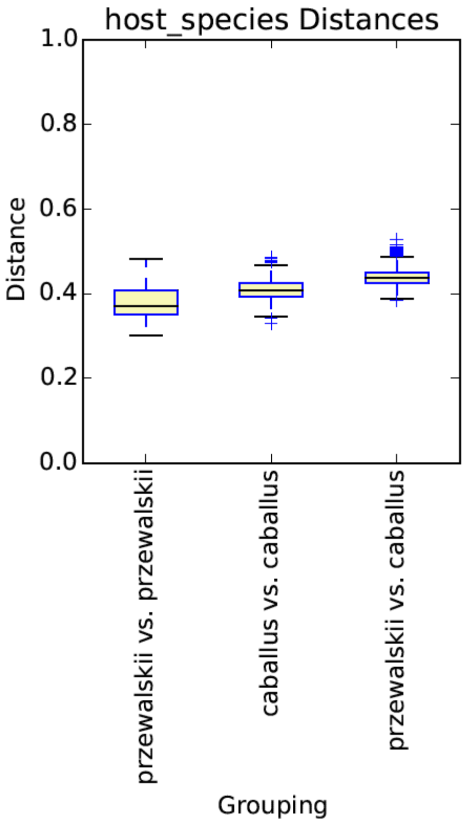


**Figure S7**. Within and between horse population distances of fecal microbiome samples were compared. Inter-individual distances of PH fecal samples are significantly smaller (microbiomes are more similar) than inter-individual distances between individuals of domestic horse (p=0.01). Both PH and domestic horse within horse population microbiome distances are significantly smaller than all between horse population distances (between species vs. within PH p=0.01, vs. within domestic p=0.01).

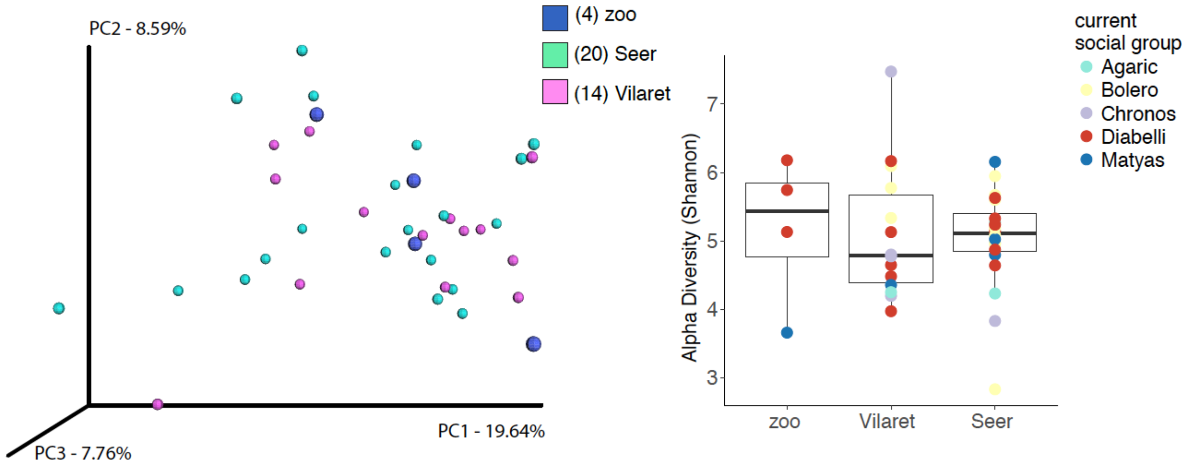


**Figure S8.** (A) PCoA plot of a Bray Curtis distance matrix of PH trnL data (samples collapsed to one representative sample per individual) ($r^2 = 0.059$, Pr(>F) = 0.286). Fecal plant diversity representing horses born in European zoos are shown as large blue spheres (B) Shannon diversity by location of birth with individual data points colored by social group (all pairwise p-values =1 after adjusting for multiple comparisons).
